# Supplementary material for: Self-shrinking soft demoulding for complex high-aspect-ratio microchannels
Source: Nat Commun. 2022 Aug 29;13:5083. doi: 10.1038/s41467-022-32859-z (PMC9424246; doi:10.1038/s41467-022-32859-z)
Supplement: Supplementary file 1 — Supplementary Information [file 41467_2022_32859_MOESM1_ESM.pdf]

Supplementary Information

Dongliang Fan[1,2], Xi Yuan[3,4], Wenyu Wu[5], Renjie Zhu[1,2], Xin Yang[1,2], Yuxuan Liao[1,2], Yunteng Ma[1], Chufan Xiao[3,4], Cheng Chen[6], Changyue Liu[3,4], Hongqiang Wang[1,2,7]*, Peiwu Qin[3,4]*

[1]Shenzhen Key Laboratory of Biomimetic Robotics and Intelligent Systems, Department of Mechanical and Energy Engineering, Southern University of Science and Technology, Shenzhen, Guangdong, 518055, China.

[2]Guangdong Provincial Key Laboratory of Human-Augmentation and Rehabilitation Robotics in Universities, Southern University of Science and Technology, Shenzhen 518055, China.

[3]Institute of Biopharmaceutical and Health Engineering, Tsinghua Shenzhen International Graduate School, Shenzhen, Guangdong, 518055, China.

[4]Center of Precision Medicine and Healthcare, Tsinghua-Berkeley Shenzhen Institute, Shenzhen, Guangdong Province, 518055, China.

[5]School of System Design and Intelligent Manufacturing, Southern University of Science and Technology, Shenzhen, Guangdong, 518055, China.

[6]Department of Biomedical Engineering, National University of Singapore, Singapore 117575, Singapore.

[7]Southern Marine Science and Engineering Guangdong Laboratory (Guangzhou), Guangzhou 510000, China.

Corresponding authors: Hongqiang Wang, wanghq6@sustech.edu.cn; Peiwu Qin, pwqin@sz.tsinghua.edu.cn

Supplementary Notes

Supplementary Note 1: Soft demoulding mechanism for the TPU filament

We built the demoulding model for the TPU filament. Due to the hyper-elastic behaviour of the TPU filament (Supplementary Fig. 4d), the relationship between peel force F_{peel} and peel angle θ can be expressed by:

$$\frac{2{F_{peel}}^2}{Ed} + \frac{F_{peel}(1 - cos\theta)}{\pi d} = \Delta E_s \qquad (1')$$

where E is Young's modulus, and d is the filament diameter.

Different from the thermoplastic resin (hot melt glue, 3748Q), the TPU filaments exhibit an increment in peel force with embedded length increasing since the higher strain was engaged during the demoulding processes (see Supplementary Fig. 4e, f). The deformed angle and applied force enlarge as the strain increases (Supplementary Fig. 4g). In addition, for larger diameters, the peel force increases, and the peel angles increase accordingly (Supplementary Fig. 4h).

Compared to rigid demoulding, an increment in peel force is also observed in the demoulding of the TPU filament with the embedded length increasing, but the applied force is much smaller. Different from the effect of the contact area on the shear force of rigid demoulding, the TPU filament demoulding still obeys the soft demoulding mechanism (see equation (1')), and the increasing peel force can be explained by the increasing strain, as shown in Supplementary Fig. 4f. Moreover, the TPU filament is reusable until it exceeds its elastic region.

Supplementary Note 2: Demoulding of the filament with a bulge

As shown in Fig. 2f, there is a limitation on the bulge size—after deformation, the bulge size should be smaller than other sections of the channel.

Assuming that the elasticity of the filament material is linear and the length of the bulge is much smaller than the filament length, for a uniform filament (Supplementary Fig. 11a), we have the Poisson's ratio:

$$\nu = -\frac{\varepsilon_{trans}}{\varepsilon_{axial}} = \frac{R - r/_R}{l - L/_L} = \frac{R - r}{R\varepsilon} \tag{2'}$$

where R is the original radius, r is the stretched radius, and ε is axial strain.

For the bulge filament (Supplementary Fig. 11b), the force is constant in every part of the filament during the stretch process:

$$F_{bulge} = F_{filament} \tag{3'}$$

Then we have:

$$\frac{\sigma_1}{\sigma_2} = \frac{{R_2}^2}{{R_1}^2} \tag{4'}$$

Since the material elasticity is linear:

$$\frac{\varepsilon_1}{\varepsilon_2} = \frac{\sigma_1}{\sigma_2} \tag{5'}$$

Combining equation (3') -(5'), we get:

$$r_2 = R_2 - \frac{{R_2}^3 \varepsilon \nu}{{R_1}^2} \tag{6'}$$

where R_1 is the original filament diameter, r_1 is the stretched filament diameter, R_2 is the original bulge diameter, and r_1 is the stretched bulge diameter.

To ensure that the bulge can pass through the microchannel, we should have:

$$R_1 \geq R_2 - \frac{{R_2}^3 \varepsilon \nu}{{R_1}^2} \qquad (7')$$

Therefore, the bulge size is limited by the original filament diameter, Poisson's ratio, and the ultimate strain of the filament.

Supplementary Note 3: Experimental results of the soft strain sensor

During the stretching process of the soft strain sensor, assuming the cross-sectional geometry of the channel remains constant, the cross-sectional area ratio A_0/A_t is calculated as follows:

$$\frac{A_0}{A_t} = (1 - \nu\varepsilon)^2 \qquad (8')$$

where A_0 is the original cross-sectional area, A_t is the cross-sectional area at a certain strain, ν is Poisson's ratio (e.g., 0.39 for Ecoflex 0030 in this work), and ε is the strain. As shown in Supplementary Fig. 6b, the resistance change $\Delta R/R$ of the stretchable conductive strain sensor is calculated by:

$$\frac{\Delta R}{R} = \frac{1 + \varepsilon}{(1 - \nu\varepsilon)^2} - 1 \qquad (9')$$

As shown in Supplementary Fig. 7c, d, the discrepancy between our strain sensor and the estimation is only 4%. It means this sensor's performance is predictable. Suppose the channel's cross section is noncircular, such as a triangle or a square. In that case, this model loses accuracy due to distortion, and the area ratio differs during stretching according to the FEA model (ABAQUS Explicit 2020, Supplementary Fig. 7d). The accuracy of the strain sensor containing the circular microchannel remains stable for the various stretching rates (Supplementary Fig. 7e). As shown in Fig. 5c, the wearable sensor was stretched, and the voltage increased as the subject's arm was bent.

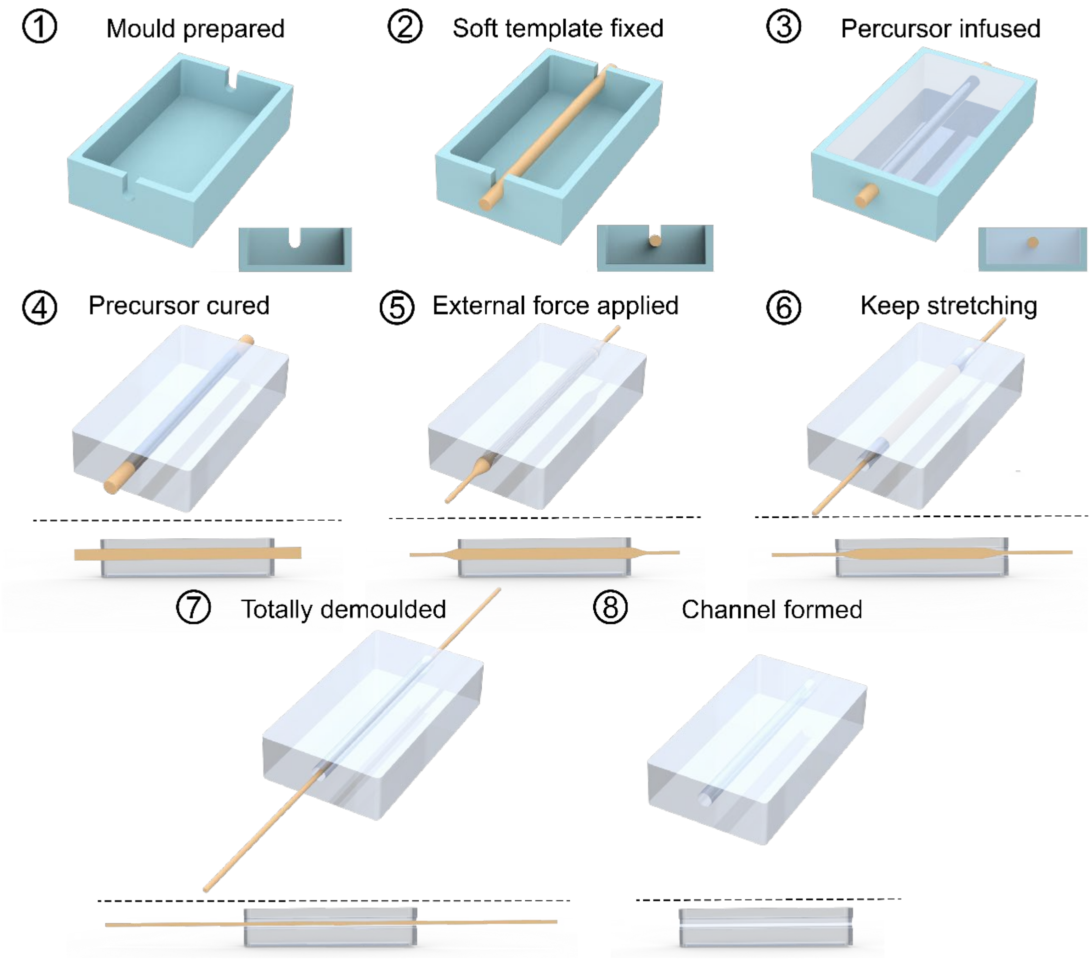

Supplementary Fig. 1 The microchannel fabrication process by soft demoulding. First, a 3D printed mould is prepared. After a soft filament is fixed at the centre of the mould, silicone precursor is poured into the mould and thermally cured. Then the cured silicone is separated from the mould. Next, the soft template is peeled out of the matrix while its two ends are stretched. Finally, a silicone matrix with a microchannel inside is produced.

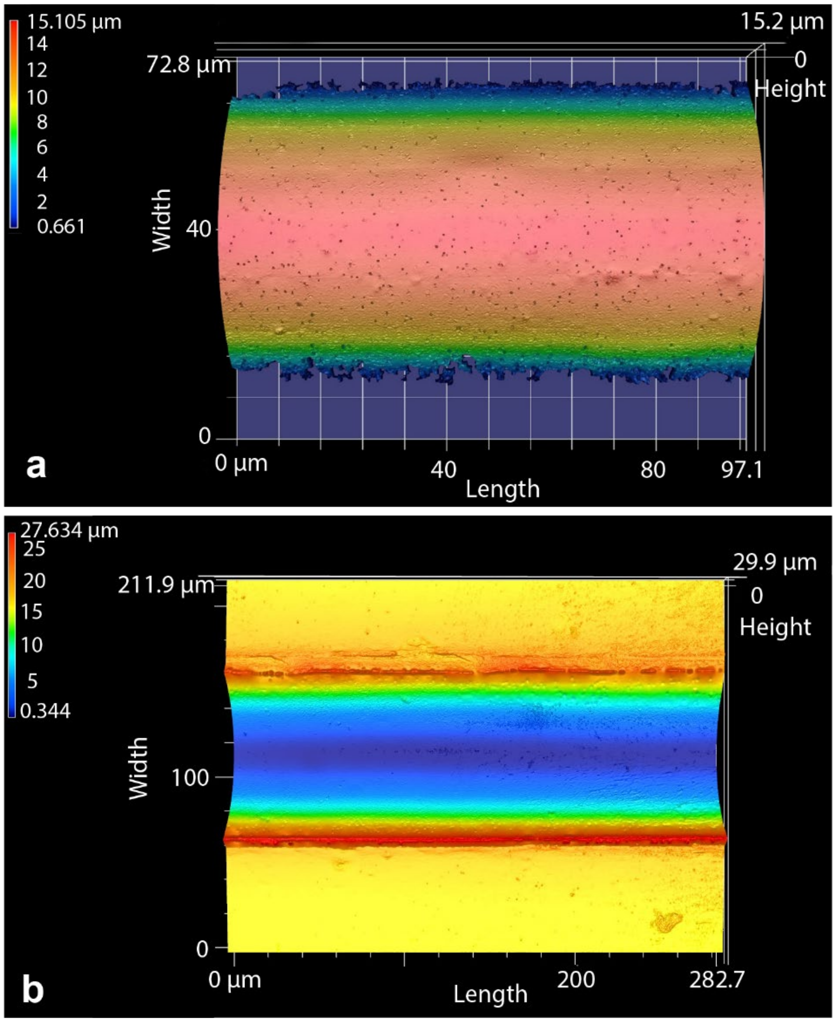

Supplementary Fig. 2 Roughness and morphology measurement of a soft template and a microchannel made by soft demoulding. a, The morphology of a thermoplastic resin filament. **b**, The morphology of a microchannel.

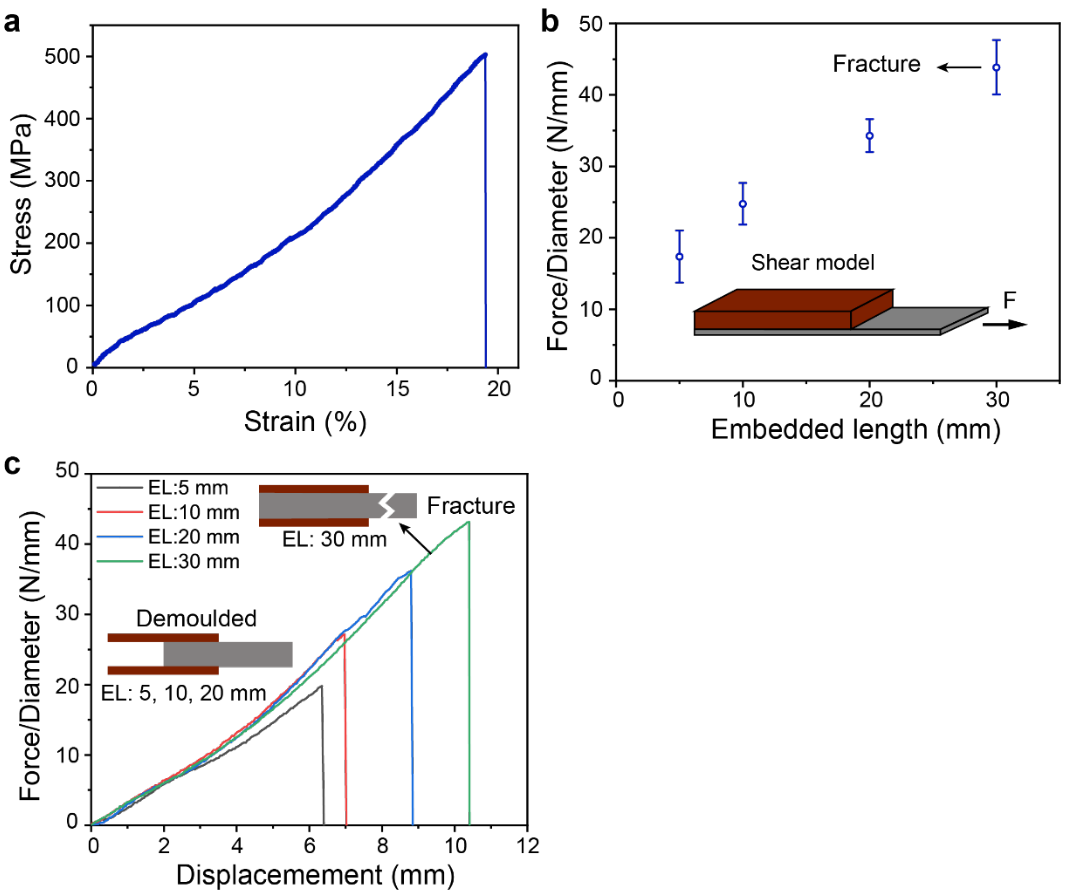

Supplementary Fig. 3 Characterization of rigid demoulding of nylon filaments. a, The stress-strain curve for a nylon filament. **b**, The relationship between pull-out force and embedded lengths (EL). The data are presented as mean values $\pm$ standard deviation for the number of trials n= 3. **c**, The pull-out force-displacement curve of nylon templates with different embedded lengths.

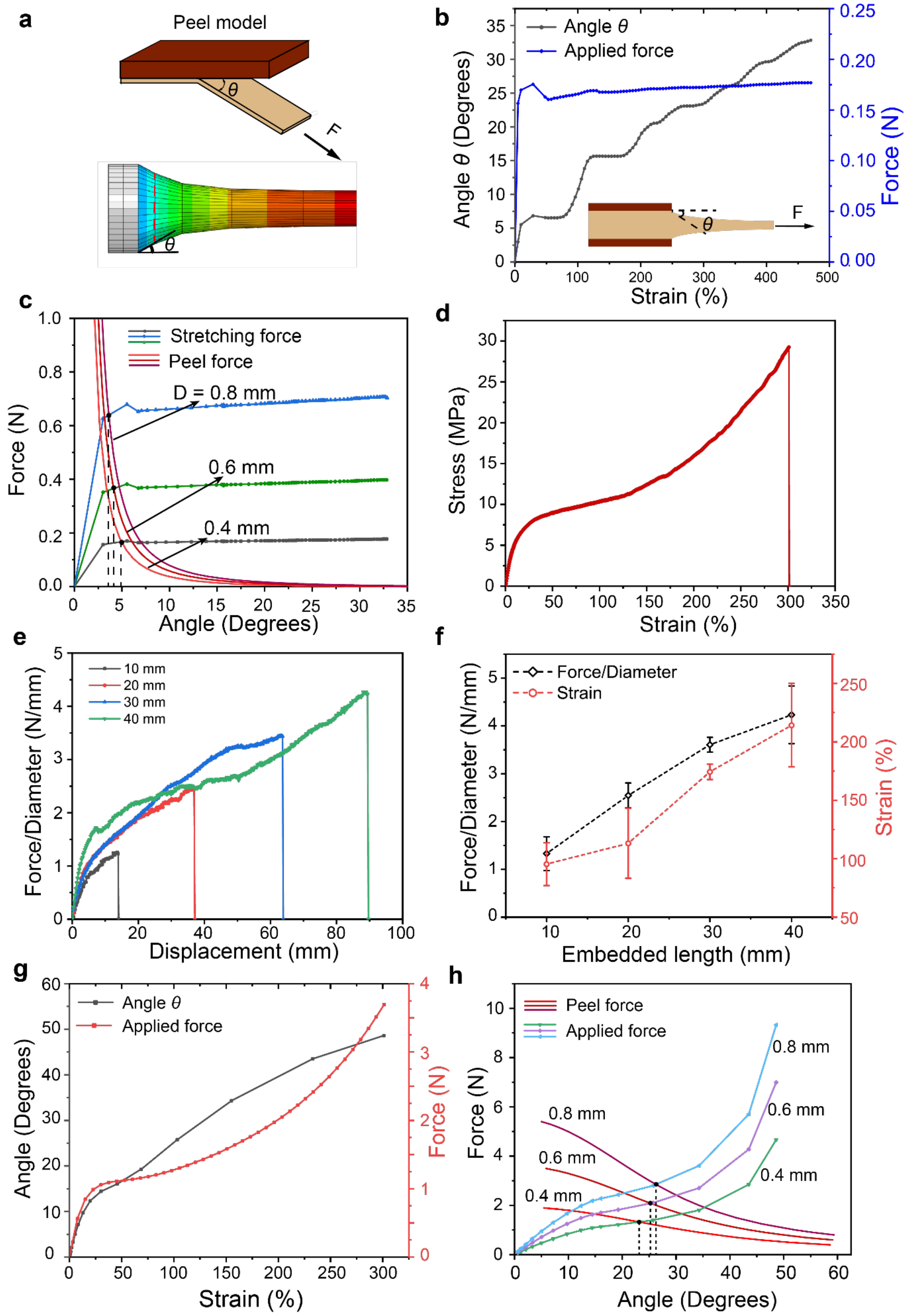

Supplementary Fig. 4 Characterization of soft demoulding. a, The peel model for soft demoulding. θ represents the peel angle. **b**, The angle θ and the stretch force changing with strain for the soft template (thermoplastic resin). **c**, The effect of soft template (thermoplastic resin) diameter on the peel force and peel angle. **d**, The stress-strain curve for a soft template (TPU). **e**, The applied force-displacement curve of TPU templates with different embedded lengths. **f**, The effect of embedded lengths of TPU filaments on the pull-out force and strain. The data are presented as mean values ± standard deviation for the number of trials n= 3. **g**, The angle θ and the stretch force changing with strain for the TPU filaments. **h**, The effect of the TPU filaments' diameter on the peel force and peel angle.

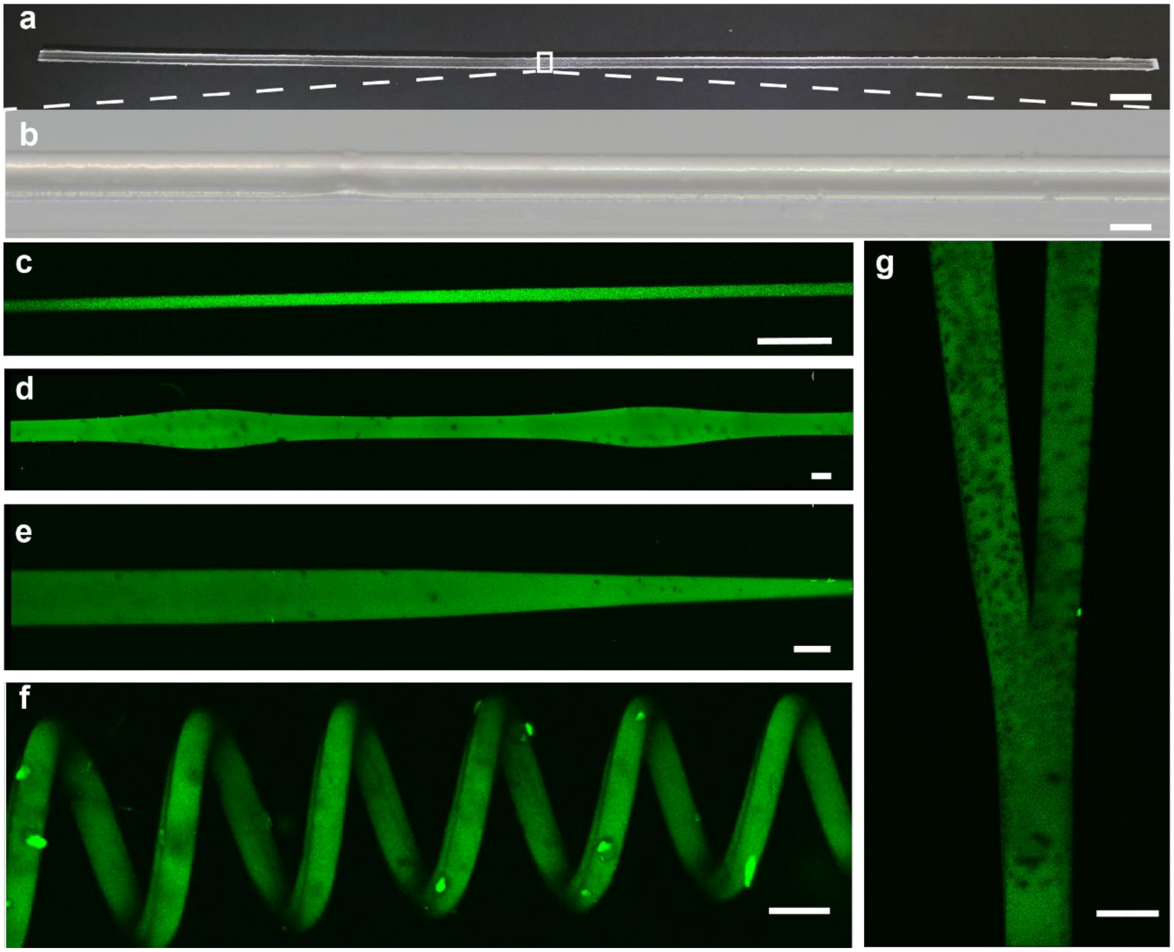

Supplementary Fig. 5 A Microchannel with super-high aspect ratio and microchannels infused with fluorescent dye. a, A microchannel with 60 cm in length and 100 µm in diameter (aspect ratio: 6000). Scale bar, 2 cm. **b**, The feature part of (a). Scale bar, 100 µm. **c**, The single straight microchannel. **d**, The microchannel with a spindle-knotted structure. **e**, The microchannel with a tapered shape. **f**, The microchannel with a helical geometry. **g**, The branched microchannel. Scale bars (c-g), 200 µm. Images are representative of 3 independent microchannels (experimental replicates).

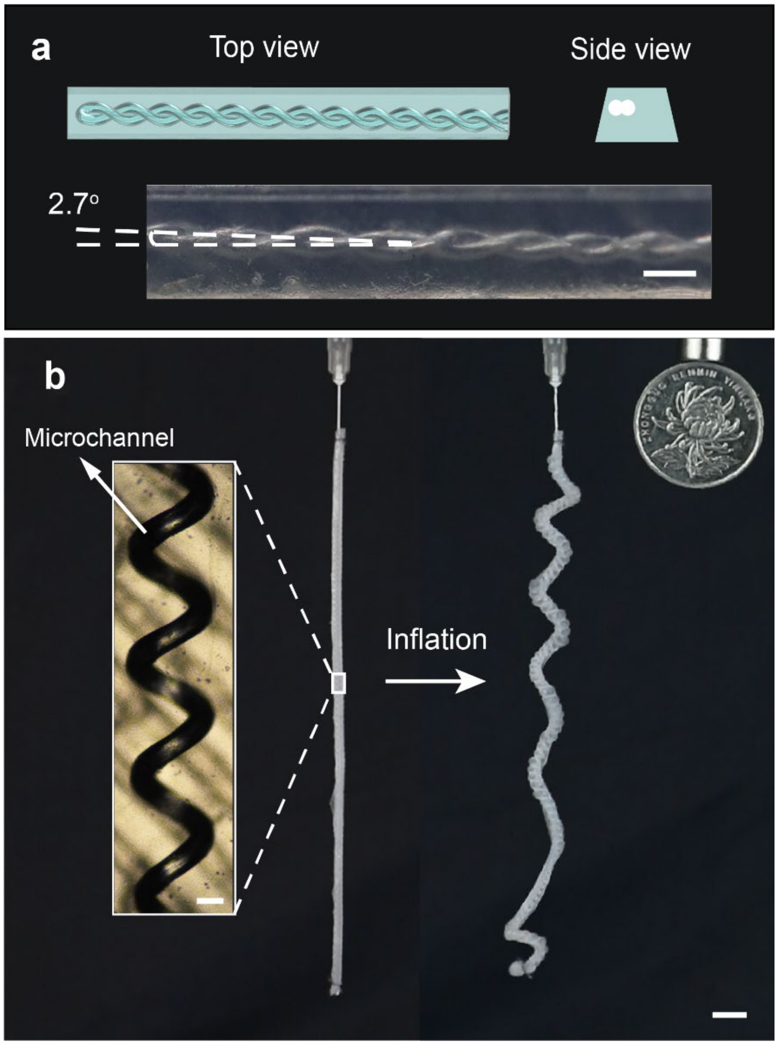

Supplementary Fig. 6 Applications of soft demoulding on soft robotics for shape morphing. **a**, The structure of the soft worm robot. Scale bar, 500 μm. **b**, The structure of the soft tendril robot and its winding state after inflation. The left inset shows the structure of the helical microchannel (scale bar: 200 μm). Scale bar, 5 mm. Images are representative of 3 independent microchannels (experimental replicates).

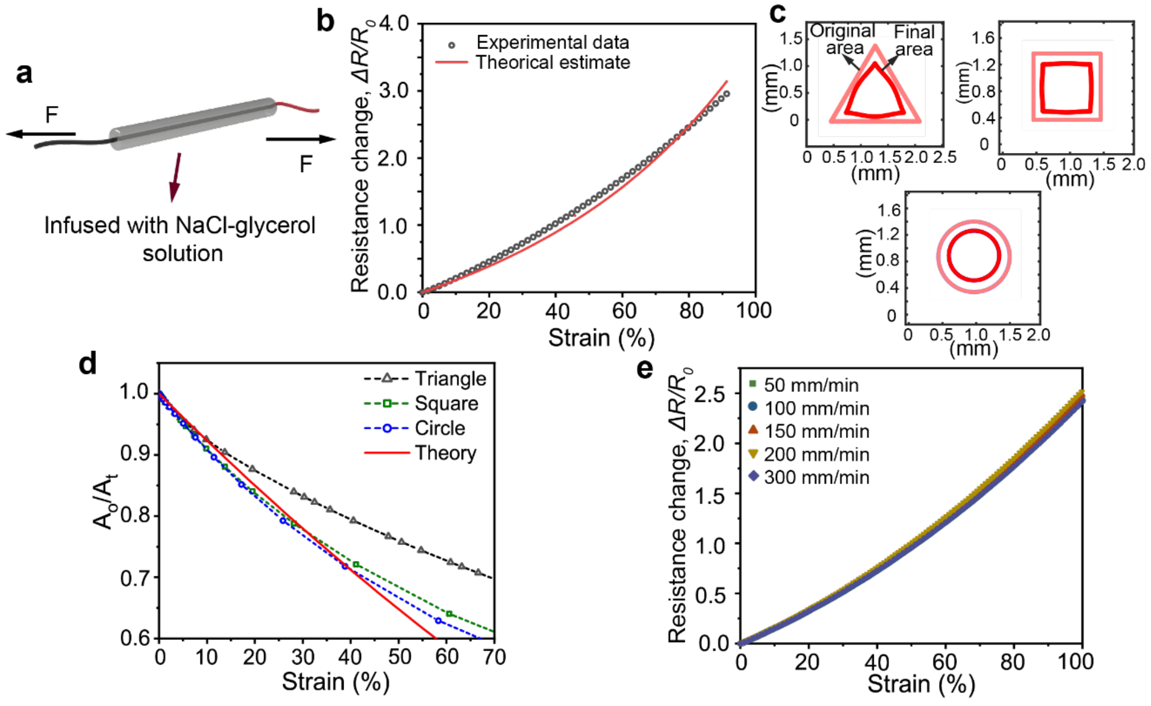

Supplementary Fig. 7 Characterization of the soft sensor. **a**, The structure of the soft stain sensor. **b**, The relationship between strain and resistance change, where ΔR is the resistance increment, and R_0 is the initial resistance. **c**, FEA results of cross sections after deformation of cross-sectional geometries (triangle, square, and circle). **d**, The response of cross sections after the strain applied. where V_0 is the initial area, and A_t is the area at corresponding strain. **e**, The effect of strain rate on the resistance change.

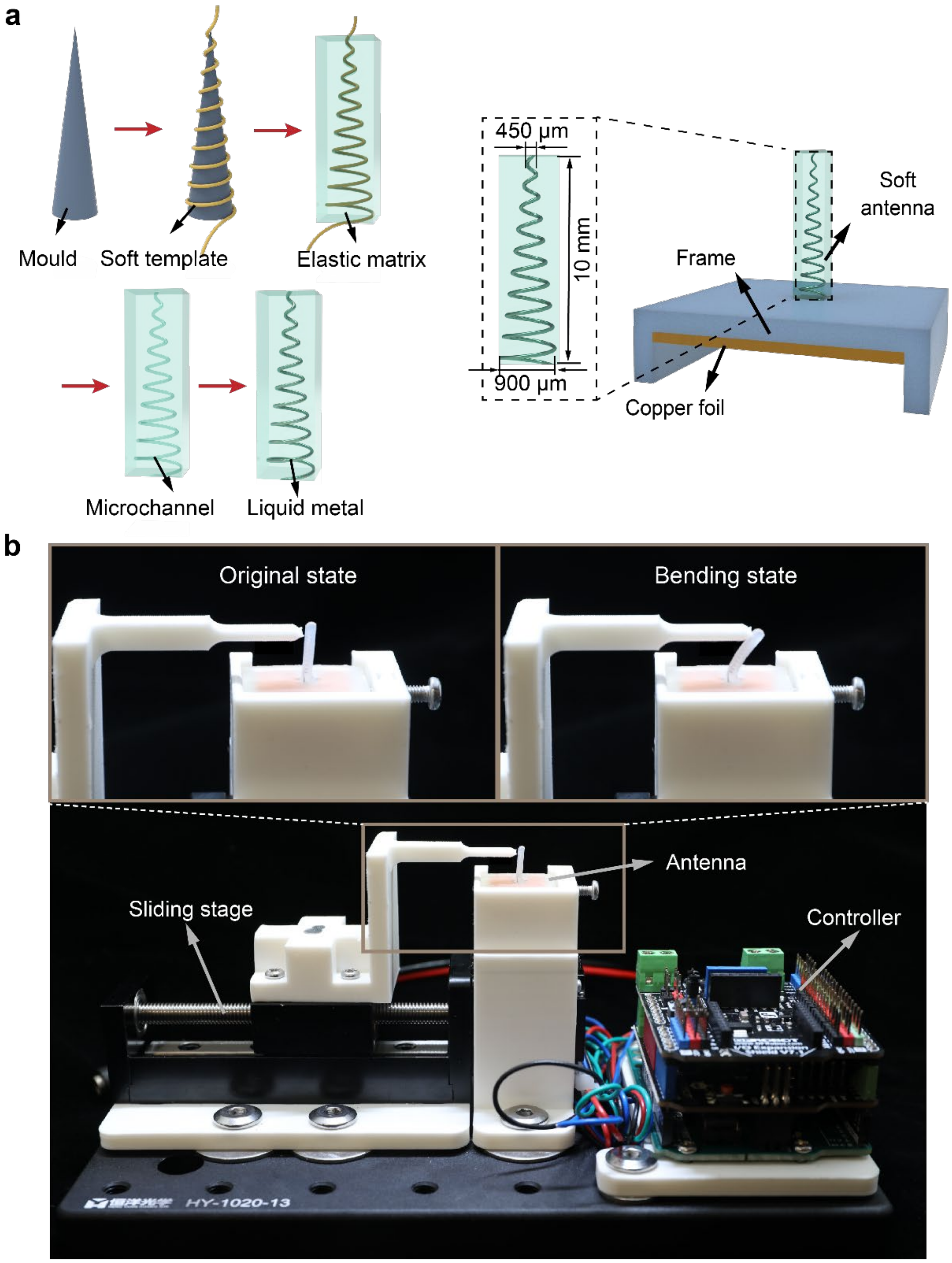

Supplementary Fig. 8 Applications of the soft antenna. a, Fabrication process of the soft antenna with a 3D helical microchannel. A 3D helical soft filament is fabricated by first aligning a straight soft filament to a metal cone into 3D geometry and fixing the helical geometry by heat treatment. Then, the soft template is immersed into the liquid precursor and thermally cured. Next, the template is demoulded from the elastic matrix. Finally, the liquid metal is injected into the helical microchannel. **b**, The setup for the reflection coefficient tests.

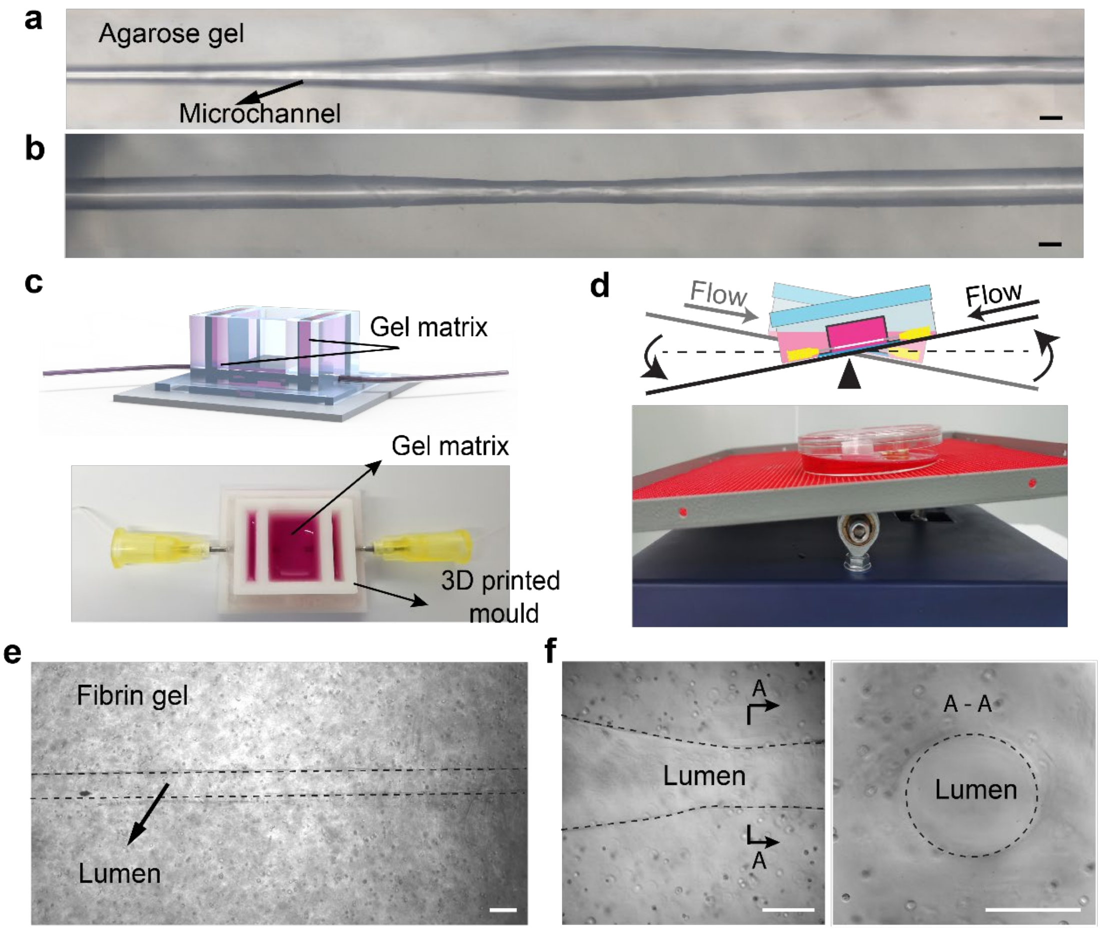

Supplementary Fig. 9 Applications on biomedical devices based on soft demoulding methodology. a, The spindle-shaped microchannel in the agarose gel (left diameter: 100 μm, the maximum diameter of the spindle shape: 450 μm, and right diameter: 200 μm). b, The straight microchannel with a narrow neck (diameter: 300 μm, and the narrow diameter: 150 μm). **c**, The schematic and real artificial vessel model. **d**, The rocking platform introducing haemodynamic flow in the cell-culturing process to create the microscale vessel. **e**, The brightfield image of the straight artificial vessels (diameter: 150 μm) in the fibrin gels formed by soft demoulding. **f**, The brightfield images of the top view and cross-sectional view of the tapered artificial vessels (the minimum diameter: 250 μm, the maximum diameter: 500 μm). Scale bars, 200 μm. Images are representative of 3 independent microchannels (experimental replicates).

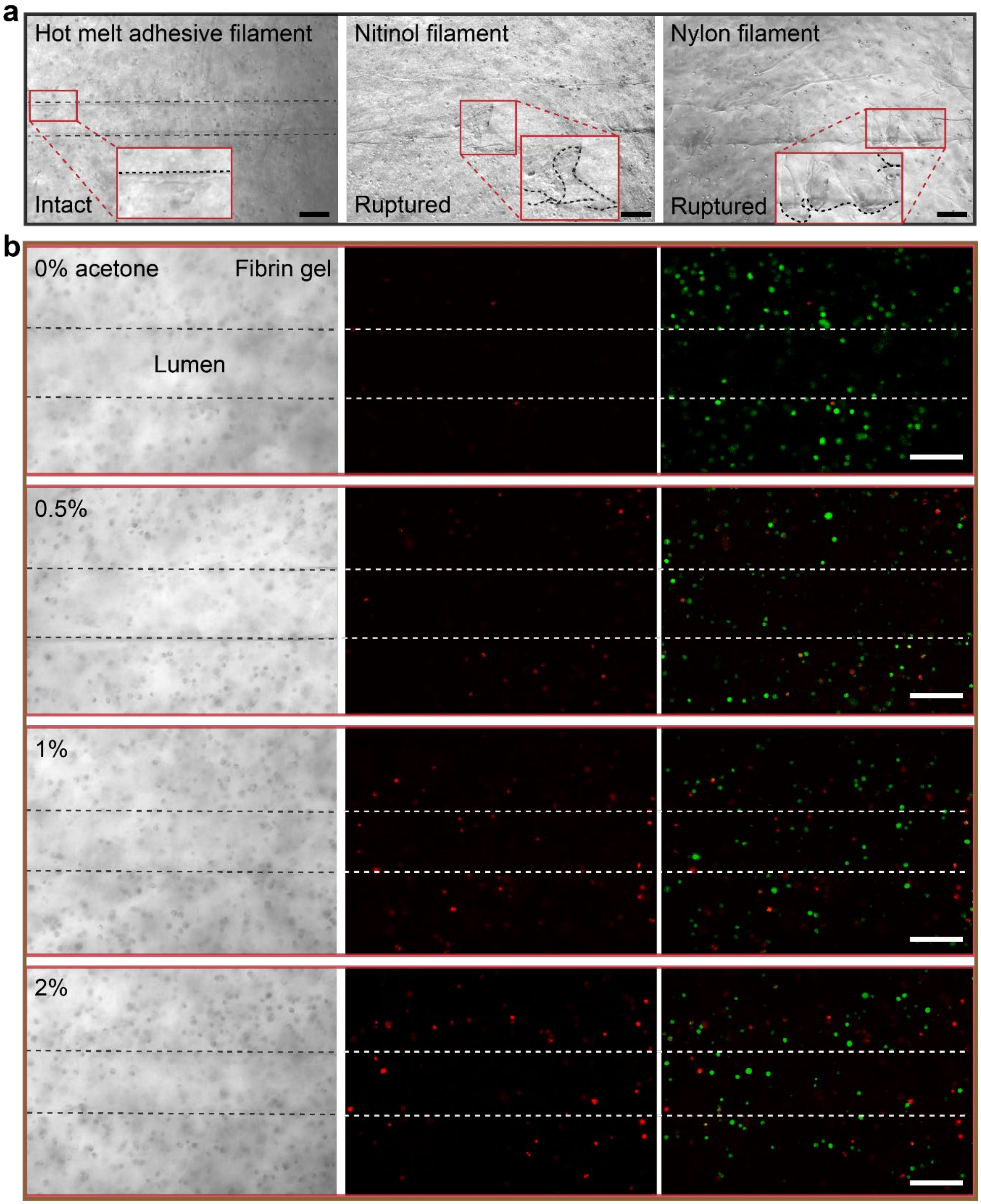

Supplementary Fig. 10 Effect of rigid demoulding and solvent resident on biomedical applications. a, The microchannels fabricated by hot melt adhesive filament (intact), Nitinol filament (ruptured), and nylon filament (ruptured) from left to right. Scale bars, 200 μm. **b**, Effect of different acetone concentrations on the death rate of cells. The left ones are brightfield images, and the middle (dead cells) and right ones (dead and live cells) are confocal images of the fibrin gel after two days of culture stained with live (green)/dead (red) essay. Scale bars, 200 μm. Images are representative of 2 independent artificial vessels (experimental replicates).

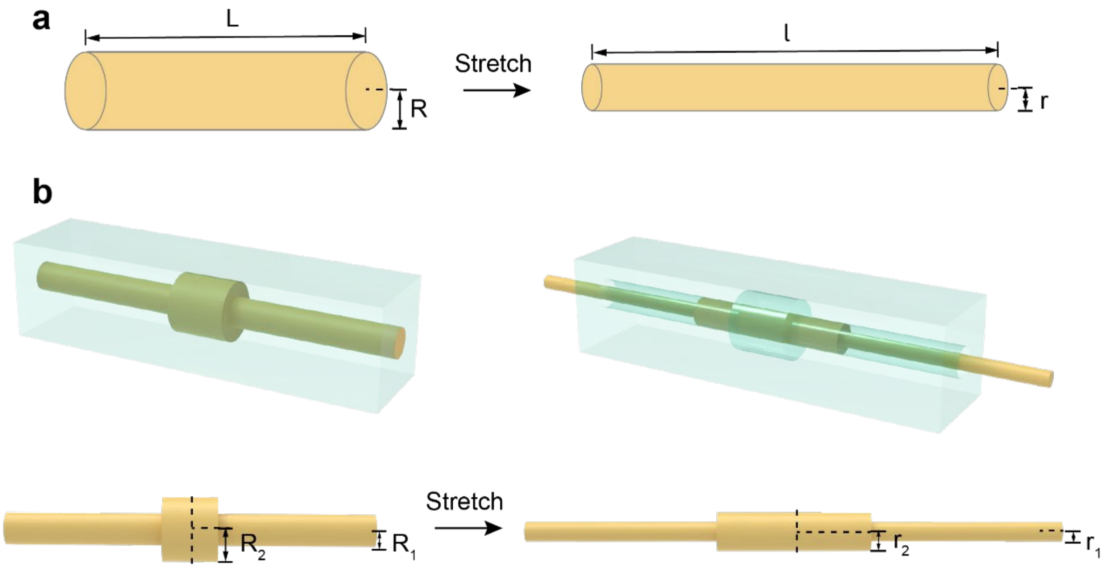

Supplementary Fig. 11 Illustrations of stretching of soft templates. **a**, Stretching of a single soft filament. **b**, Stretching of a single soft filament with a bulge.

Supplementary Table 1. Comparisons of existing microchannel fabrication technologies.

Fabrication method	**Soft lithography**	**Physical extraction (Matrix swollen and direct pulling)**	**Template dissolution**	**3D printing (Direct printing channels)**	**Soft demoulding**
Cross-sectional geometry	Rectangular shape	Round shape	Round shape	Round shape	Round shape
Feature size	10 μm[19]	10 μm[23] (matrix swollen) 100 μm[7] (direct pulling)	10 μm[27]	200 μm[17], 100 μm[20]	10 μm
Space complexity	High 2D complexity	Medium 3D complexity	High 3D complexity	High 3D complexity	High 3D complexity
Aspect ratio	N/A	629[9]	N/A	N/A	6000
Solvent-required	No	Yes	Yes	No	No
Channel surface roughness	High smooth	High smooth	High smooth	Relative rough	High smooth
Fabrication duration	Several hours	Several hours	Half-day-Several days	Several hours	Several hours
