# Supplementary material for: Self-shrinking soft demoulding for complex high-aspect-ratio microchannels
Source: Nat Commun. 2022 Aug 29;13:5083. doi: 10.1038/s41467-022-32859-z (PMC9424246; doi:10.1038/s41467-022-32859-z)
Supplement: Supplementary file 3 — Description of Additional Supplementary Files [file 41467_2022_32859_MOESM3_ESM.pdf]

Description of Additional Supplementary Files

Supplementary Movie 1

Demonstration of rigid demoulding and soft demoulding process. This video shows the difference between the conventional rigid demoulding and soft demoulding. Both a rigid template and a soft template are used to lift a weight. The rigid template (copper wire, 80 μm) successfully lifted a 500 g weight but failed to pull out of a PDMS matrix and ruptured. The soft template (thermoplastic resin, 150 μm) failed to lift a 50 g weight. During the lifting process, the soft template kept stretching till ruptured. In contrast, the soft template demoulded from the PDMS matrix successfully.

Supplementary Movie 2

Actuation of the soft worm robot. This video shows the soft robot with designed microscale airways inside was twisted to mimic the behavior of worms in defence state when pressure was applied.

Supplementary Movie 3

Actuation of the soft tendril robot. This video shows the soft robot transformed into a helical geometry when pressure was applied, and then, after a standing beam was installed, the soft robot winded around the beam just like a tendril.

Supplementary Movie 4

The FEA result of the cross-sectional geometry changing during the elastic tube stretching. The video shows the deformation of elastic tubes with different cross sections (triangle, square, and circle) under uniaxial stretching. The side view and cross-sectional view were shown in the video.

Supplementary Movie 5

Elbow bending test for the thread-like soft strain sensor. The video shows that the elbow bending behavior was detected by the slender, soft strain sensor woven in a wearable sleeve, and the voltage signals were shown on the display screen.
