# Supplementary material for: Self-shrinking soft demoulding for complex high-aspect-ratio microchannels
Source: Nat Commun. 2022 Aug 29;13:5083. doi: 10.1038/s41467-022-32859-z (PMC9424246; doi:10.1038/s41467-022-32859-z)
Supplement: Supplementary file 9 — Reporting Summary [file 41467_2022_32859_MOESM9_ESM.pdf]

Corresponding author(s): Hongqiang WANG

Last updated by author(s): Aug 16, 2022

Reporting Summary

Nature Portfolio wishes to improve the reproducibility of the work that we publish. This form provides structure for consistency and transparency in reporting. For further information on Nature Portfolio policies, see our Editorial Policies and the Editorial Policy Checklist.

Statistics

For all statistical analyses, confirm that the following items are present in the figure legend, table legend, main text, or Methods section.

n/a	Confirmed	
☐	☒	The exact sample size (*n*) for each experimental group/condition, given as a discrete number and unit of measurement
☐	☒	A statement on whether measurements were taken from distinct samples or whether the same sample was measured repeatedly
☒	☐	The statistical test(s) used AND whether they are one- or two-sided<br>*Only common tests should be described solely by name; describe more complex techniques in the Methods section.*
☒	☐	A description of all covariates tested
☒	☐	A description of any assumptions or corrections, such as tests of normality and adjustment for multiple comparisons
☐	☒	A full description of the statistical parameters including central tendency (e.g. means) or other basic estimates (e.g. regression coefficient) AND variation (e.g. standard deviation) or associated estimates of uncertainty (e.g. confidence intervals)
☒	☐	For null hypothesis testing, the test statistic (e.g. *F*, *t*, *r*) with confidence intervals, effect sizes, degrees of freedom and *P* value noted<br>*Give P values as exact values whenever suitable.*
☒	☐	For Bayesian analysis, information on the choice of priors and Markov chain Monte Carlo settings
☒	☐	For hierarchical and complex designs, identification of the appropriate level for tests and full reporting of outcomes
☒	☐	Estimates of effect sizes (e.g. Cohen's *d*, Pearson's *r*), indicating how they were calculated

Our web collection on statistics for biologists contains articles on many of the points above.

Software and code

Policy information about availability of computer code

Data collection	Mechanical testing data were collected by using MTS TW Elite software (version 4.5.2.423). Voltage data during sensor tests were collected by using LabVIEW 2019. Imaging data were collected by using Keyence image-acquisition software (version 1.1.3.184) and Nikon image-acquisition software NIS-Elements AR (version 5.11.00). Simulation of soft templates and microchannels were performed in ABAQUS Explicit 2020.The code in this study has been deposited in the Code Ocean repository (https://codeocean.com/capsule/7726423/tree/v1) or can be requested from the corresponding authors.
Data analysis	Statistical analysis and plotting were performed in Origin 2018, and MATLAB 2019a.

For manuscripts utilizing custom algorithms or software that are central to the research but not yet described in published literature, software must be made available to editors and reviewers. We strongly encourage code deposition in a community repository (e.g. GitHub). See the Nature Portfolio guidelines for submitting code & software for further information.

Data

Policy information about availability of data

All manuscripts must include a data availability statement. This statement should provide the following information, where applicable:

- Accession codes, unique identifiers, or web links for publicly available datasets
- A description of any restrictions on data availability
- For clinical datasets or third party data, please ensure that the statement adheres to our policy

The data that support the findings of this study are available within the paper and its Supplementary Information and from the corresponding author upon request.

Field-specific reporting

Please select the one below that is the best fit for your research. If you are not sure, read the appropriate sections before making your selection.

- [x] Life sciences
- [ ] Behavioural & social sciences
- [ ] Ecological, evolutionary & environmental sciences

For a reference copy of the document with all sections, see nature.com/documents/nr-reporting-summary-flat.pdf

Life sciences study design

All studies must disclose on these points even when the disclosure is negative.

Sample size	Since this study involved the initial demonstration of new methodology, sample sizes were thus selected to ensure confidence in the reproducibility of the methodology. Based on other similar studies in the field, the microchannel formation and artificial vessel experiments were independently performed at least three times (Kinstlinger, I.S., et. al, Nat. Biomed. Eng., 4, 916–932 (2020)). For experiments to investigate the effect of solvent residents on biomedical applications, two replicates were performed since the results from these two replicates were highly consistent and did not require statistical analysis.
Data exclusions	For artificial vessel experiments, the gels were excluded from analysis once the channels were raptured or collapsed during the cell culturing processes.
Replication	For microchannel formation in the biocompatible matrix (agarose) by soft demoulding, more than three replicates with microchannels inside were successfully fabricated. For the rigid demoulding experiment, three biological replicates were performed, and the results were consistent. For artificial vessel experiments, three artificial vessels were independently and successfully fabricated, demonstrating the reproducibility of the soft demoulding method. To study the effect of solvent resident on biomedical applications, two replication was performed and got similar results.
Randomization	No specific method of randomization was used to generate microchannels or in cell experiments. Microchannels are designed purposely, and the goal is to create the expected shape of the microchannel, which cannot be randomized. In artificial vessel experiments, the individual cell was proliferated from cell clones and genetically identical. Thus, there is no point to randomize identical experiments.
Blinding	Blinding was not relevant in this study during the data collecting process since the conclusion is not dependent on statistical inference. Imaging data collection aims to demonstrate representative cell morphology in a microchannel, which requires parameter optimization and a decent field of view selection. The sample was visualized under microscopy, and random regions of interest were collected with blinding. The investigators were not blinded to group allocation during data analysis, which is irrelevant to this study since the presence or absence of cell survival is important for drawing the conclusion.

Reporting for specific materials, systems and methods

We require information from authors about some types of materials, experimental systems and methods used in many studies. Here, indicate whether each material, system or method listed is relevant to your study. If you are not sure if a list item applies to your research, read the appropriate section before selecting a response.

Materials & experimental systems

n/a	Involved in the study	
x		Antibodies
	x	Eukaryotic cell lines
x		Palaeontology and archaeology
x		Animals and other organisms
x		Human research participants
x		Clinical data
x		Dual use research of concern

Methods

n/a	Involved in the study	
x		ChIP-seq
x		Flow cytometry
x		MRI-based neuroimaging

Eukaryotic cell lines

Policy information about cell lines

Cell line source(s)	Human umbilical vein endothelial cells (HUVECs) were purchased from Lonza (catalog number: CC-2517); BALB/C 3T3 cell was obtained from the Cell Resource Center, Peking Union Medical College (resource number: 1101MOU-PUMC000186).
Authentication	HUVEC cell line was authenticated with STR profiling by the supplier (Lonza). The identity of the BALB/C 3T3 cell line was authenticated with STR profiling (FBI, CODIS) by Cell Resource Center, Peking Union Medical College. No further authentication was performed.
Mycoplasma contamination	Cell lines were not tested for mycoplasma contamination.
Commonly misidentified lines (See ICLAC register)	No commonly misidentified cell lines were used.
